# Drug Susceptibility and Molecular Epidemiology of *Klebsiella pneumoniae* Bloodstream Infection in ICU Patients in Shanghai, China

**DOI:** 10.3389/fmed.2021.754944

**Published:** 2021-10-13

**Authors:** Shuzhen Xiao, Tianchi Chen, Hairu Wang, Qian Zeng, Qing Chen, Zhitao Yang, Lizhong Han, Erzhen Chen

**Affiliations:** ^1^Department of Laboratory Medicine, Ruijin Hospital, Shanghai Jiao Tong University School of Medicine, Shanghai, China; ^2^Department of Clinical Microbiology, Ruijin Hospital, Shanghai Jiao Tong University School of Medicine, Shanghai, China; ^3^Department of Laboratory Medicine, Renji Hospital, Shanghai Jiao Tong University School of Medicine, Shanghai, China; ^4^Departments of Clinical Laboratory, Shanxi Provincial People's Hospital, Affiliated of Shanxi Medical University, Taiyuan, China; ^5^Emergency Department, Ruijin Hospital, Shanghai Jiao Tong University School of Medicine, Shanghai, China

**Keywords:** bloodstream infection, *Klebsiella pneumoniae*, intensive care units, drug susceptibility, molecular epidemiology

## Abstract

**Background:** Bloodstream infections (BSIs) are recognized as important nosocomial infections. *Klebsiella pneumoniae* is one of the major causes of bacteremia. This retrospective study focused on drug susceptibility and molecular epidemiology of *K. pneumoniae* isolated from intensive care unit (ICU) patients with BSI in Shanghai, China.

**Methods:** Consecutive *K. pneumoniae* isolates were collected from ICU patients. Antibiotic susceptibility testing was conducted by the broth microdilution method. PCR was performed to detect antimicrobial resistance genes. We also completed multilocus sequence typing (MLST) and GoeBURST was used to analyze the result of MLST.

**Results:** A total of 78 *K. pneumoniae* isolates were enrolled. *K. pneumoniae* from ICU-BSIs were highly resistant to almost all common antibiotics. The most frequent resistance determinants responsible for extended-spectrum β-lactamase (ESBL) producers were *bla*_CTX−M−14_, *bla*_CTX−M−15_, and *bla*_CTX−M−55_. KPC was the only enzyme, which was detected by the carbapenemase producers. The most principal sequence types (STs) were ST11, ST15, and ST23.

**Conclusion:** This study presents for the first time the antibiotic resistance phenotype and molecular epidemiology of *K. pneumoniae* isolated from ICU patients with BSIs in Shanghai. ICU-BSI *K. pneumoniae* is characteristic of a high resistance rate. The occurrence of the KPC-2 enzyme may result from nosocomial clonal dissemination of ST11 *K. pneumoniae*.

## Introduction

Bloodstream infection (BSI) is a leading cause of morbidity and mortality in both children and adults worldwide (1–3). Patients in intensive care units (ICUs) have a high risk to suffer from BSIs, due to the high severity of disease, the reduced host defenses, frequent use of invasive medical devices, and inadequate infection control procedures (4–6). BSIs occur in 5–15% of all patients within the first month of hospitalization in ICU (7). Meanwhile, the outcome of these infections is often poor, with an attributable mortality of up to 70% (8).

*Klebsiella pneumoniae*, an environmental or opportunistic pathogen, is frequently associated with HAIs and is recognized as the major source of BSIs caused by Gram-negative bacteria all over the world (6, 9, 10). According to European Center for Disease Prevention and Control, *K. pneumonia* ranked third in the main causative microorganisms of ICU-BSI (11). The SENTRY Antimicrobial Surveillance Program demonstrated that the prevalence of BSI *K. pneumoniae* increased consistently between 1997 and 2016 (12). Consistent with these results, *K. pneumoniae* is the second most common bacteremia pathogen in China, based on a retrospective survey involving 10 cities (13). Unfortunately, alongside the high frequency, *K. pneumoniae* can also develop antibiotic resistance, with resistance to β-lactams being most clinically significant (9). In China, *K. pneumoniae* was the most predominant pathogen in carbapenem-non-susceptible Enterobacteriaceae and the resistance rate of *K. pneumoniae* against carbapenem enhanced remarkably (from 3.0 to 25% in imipenem and from 2.9 to 26.3% in meropenem) (13, 14). The carbapenem-non-susceptible *K. pneumoniae*, likely to be associated with the production of carbapenemase, tend to have extensive drug-resistant (XDR, susceptibility limited to ≤ 2 categories) phenotypes, leading to limited treatment options, and poor outcomes (6, 15).

Appropriate and in-time antibiotic therapy, which depends on epidemiology characteristics and drug susceptibility profiles, is critically important to the outcome of BSIs (16). Although *K. pneumoniae* BSI in ICU patients is mortal, there are only some studies that focus on BSIs caused by *K. pneumoniae* and on sequence type (ST)11. Relatively few studies have attempted to conduct full-scale susceptibility surveillance and molecular epidemiology investigation of *K. pneumoniae* causing ICU-BSIs. In this study, we carried out the drug susceptibility testing, explored the distribution of antibiotic resistance genes, and analyzed the predominant STs of *K. pneumoniae* from ICU patients with BSIs in Shanghai. To our knowledge, this is the first study to investigate such molecular epidemiological data in mainland China. Such epidemiological data are useful to provide evidence for the empirical therapy and develop strategies to prevent these serious infections.

## Materials and Methods

### Setting and Study Design

This retrospective and cross-sectional study of *K. pneumoniae* BSI in ICU patients, aiming to analyze drug susceptibility and molecular epidemiology of this pathogen, was performed in Ruijin Hospital Affiliated with Shanghai Jiaotong University School of Medicine. It is a 3,000-bed comprehensive tertiary hospital located in Shanghai, a metropolitan region in China, with ~115,000 patient visits per year. Consecutive ICU patients with *K. pneumoniae* BSI were identified in the laboratory database of the Department of Clinical Microbiology from January 2016 to December 2019. Only the first positive blood culture of each patient was recorded and enrolled in follow-up experiments.

This study was approved by the Ethics Committee of Ruijin Hospital affiliated with Shanghai Jiaotong University School of Medicine. The Review Board waived the request for informed consent because our study only put emphasis on bacteria and exerted no effect on patients.

### Microbiology Identification and Storage

Identification of isolates from ICU patients with BSI was conducted on matrix-assisted laser desorption ionization time of flight mass spectrometer (bioMérieux, Marcy-l'Étoile, France) (17). *K. pneumoniae* strains were stored in LB broth (Sangon Biotech, Shanghai, China) with 30% glycerol (Sangon Biotech, Shanghai, China) at −80°C for further experiments.

### Antibiotic Susceptibility Testing and Screening Test for Extended-Spectrum β-Lactamases and Carbapenemases

Drug susceptibility was examined by the broth microdilution method using Sensititre™ GNX2F (Thermo Fisher Scientific, Waltham, MA, USA). Nineteen various antibiotics were involved in the trial, including amikacin, aztreonam, cefepime, cefotaxime, ceftazidime, ciprofloxacin, colistin, doxycycline, ertapenem, gentamicin, imipenem, levofloxacin, meropenem, minocycline, piperacillin/tazobactam, ticarcillin-clavulanic acid, tigecycline, tobramycin, and trimethoprim/sulfamethoxazole. *Pseudomonas aeruginosa* ATCC 27853, *K. pneumoniae* ATCC 700603, and *Escherichia coli* ATCC 25922 were used as quality control in the antibiotics susceptibility assay. Results were interpreted according to Clinical and Laboratory Standards Institute (CLSI) criteria (18). For tigecycline, the result was interpreted based on European Committee on Antimicrobial Susceptibility Testing (EUCAST) criteria.

Screening test for extended-spectrum β-lactamase (ESBL) production was accomplished with ceftazidime and cefotaxime according to CLSI 2019, whereas imipenem, ertapenem, or meropenem was used to screen for carbapenemase production (18). Screening tests for ESBL and carbapenemase production were performed by broth microdilution, and the synergy test (ceftazidime, cefotaxime, ceftazidime-clavulanate, and cefotaxime-clavulanate) was used as a confirmatory test for ESBL producers.

According to the consequences of susceptibility tests, *K. pneumoniae* isolates were classified into multidrug resistance (MDR, non-susceptibility to ≥1 agent in ≥3 antimicrobial categories), XDR, and pan-drug resistance (PDR, non-susceptibility to all agents in all antimicrobial categories).

### DNA Extraction, Detection of Resistance Genes, and Confirmation Test for ESBLs and Carbapenemases

To obtain sample DNA from *K. pneumoniae*, bacteria were resuspended in distilled water and boiled at 100°C for 15 min in order to lyse the cells and release DNA into an aqueous phase. The sample DNA was then segregated from cell fragments through centrifugation at 3,000 × *g* for 15 min and *K. pneumoniae* DNA was dissolved in the supernatant, which would be used as the origin of template DNA in the PCR analysis. We detected the genotype of *K. pneumoniae* whose result was positive in the previous screening phase to clarify the causative genes that result in their ESBL and/or carbapenemase production. Seven genes associated with ESBLs were amplified using T100 Thermal Cycler (Bio-Rad, Hercules, CA, USA), including *bla*_TEM_, *bla*_SHV_, CTX-M-1,−2,−8,-9,−25 group, and OXA-1,−2,−10 group, *bla*_VEB_, *bla*_GES_, *bla*_PER_, together with several carbapenemase genes, such as *bla*_VIM_, *bla*_IPM_, *bla*_KPC_, *bla*_GIM_, *bla*_SPM_, *bla*_SIM_, *bla*_OXA−48_, and *bla*_NDM_. After the PCR amplification, the target products were separated through electrophoresis in 1% agarose gel. All positive products were sequenced using the ABI3730xl DNAAnalyzer by Sangon Biotech (Shanghai, China). In addition, the genotypes were determined by comparing the sequencing results with the sequences in GenBank (http://www.ncbi.nlm.nih.gov/BLAST).

### Multilocus Sequence Typing

Multilocus sequence typing (MLST) of *K. pneumoniae* was based on seven conserved housekeeping genes (*gapA, infB, mdh, pgi, phoE, rpoB*, and *tonB*), which were amplified and sequenced in the study. After being aligned with the MLST database (http://bigsdb.web.pasteur.fr/klebsiella/primers_used.html), each housekeeping gene sequence was associated with a unique allele, and the combination of seven alleles determines the ST for each *K. pneumoniae* isolate. New alleles and STs discovered in our study were submitted to the curator of the database (klebsiellaMLST@pasteur.fr). GoeBURST was used for the MLST analysis, demonstrating the allelic relationship and prevalence of various STs. In this study, isolates were classified into the same group if six of the seven alleles were homologous.

### Statistical Analysis

IBM SPSS 25.0 (IBM Corp., Armonk, NY, USA) was used for statistical analysis. Values of categorical variables were presented as a percentage of the group where they were derived. The chi-square test or Fisher's exact test was utilized to compare the classified variables, as appropriate. The *p*-value < 0.05 was considered to be statistically significant.

## Results

### Characteristics of Total Patient Population

From January 2016 to December 2019, a 48-month study period, a total of 78 consecutive *K. pneumoniae* isolates were consecutively collected from ICU patients with BSI. Among them, 18 isolates were obtained in 2016, 12 in 2017, 22 in 2018, and 26 in 2019. More men (54/78) than women (24/78) were enrolled in the study, and the age of the 78 patients ranged from 17 to 91 years, with a median age of 60 years.

### Antimicrobial Susceptibility Tests

The rates of antibiotics resistance among 78 *K. pneumoniae* were displayed in Table 1. The highest resistance rates of *K. pneumoniae* isolated from ICU patients with BSI were ticarcillin-clavulanic acid (93.5%), aztreonam (93.5%), ciprofloxacin (92.3%), and levofloxacin (92.3%). In contrast, relatively low resistance appeared in colistin (11.5%) and tigecycline (23.0%). Among 78 *K. pneumoniae* isolates, 58 (74.4%) and 56 (71.7%) isolates were confirmed as ESBL-producers and carbapenemase-producers through gene detection, respectively. The ESBL-producing isolates exhibited statistically higher resistance to most antibiotics than non-ESBL-producing ones (*p* < 0.05), except for amikacin, doxycycline, minocycline, and tigecycline (*p*-value was 0.05, 0.170, 0.389, and 0.251, respectively). Similar to ESBL-producers, carbapenemase-producers exhibited statistically higher resistance to most antibiotics than non-carbapenemase-producing ones (*p* < 0.05), except for doxycycline, minocycline, trimethoprim-sulfamethoxazole, tigecycline, and colistin (*p*-value was 0.894, 0.777, 0.096, 0.394, and 0.289, respectively). The rate of XDR, MDR, and PDR *K. pneumoniae* isolates was 29.5, 61.5, and 2.6%, respectively.

**Table 1:** Rates of antibiotics resistance among *K. pneumoniae* bloodstream infections (BSIs) in intensive care unit (ICU) patients.

**Antimicrobial agents**	**Number of isolates (%)**			***p***	**Number of isolates (%)**		***p***
	**Total**	**ESBL**	**Non-ESBL**		**Carbapenemase**	**Non-carbapenemase**	
	**(***n*** = 78)**	**(***n*** = 58)**	**(***n*** = 20)**		**(***n*** = 56)**	**(***n*** = 22)**	
Ceftazidime	62 (79.4)	58 (100)	4 (20.0)	<0.0001	55 (98.2)	7 (31.8)	<0.0001
Cefotaxime	63 (80.7)	58 (100)	5 (25.0)	<0.0001	56 (100)	7 (31.8)	<0.0001
Cefepime	62 (79.4)	57 (98.3)	5 (25.0)	<0.0001	56 (100)	6 (27.3)	<0.0001
Amikacin	67 (85.8)	53 (91.3)	14 (70.0)	0.018	52 (92.8)	15 (68.1)	0.005
Gentamicin	70 (89.7)	56 (96.6)	14 (70.0)	0.001	55 (98.2)	15 (68.1)	<0.0001
Tobramycin	70 (89.7)	56 (96.6)	14 (70.0)	0.001	55 (98.2)	15 (68.1)	<0.0001
Aztreonam	73 (93.5)	57 (98.3)	16 (80.0)	0.004	56 (100)	17 (77.2)	<0.0001
Ciprofloxacin	72 (92.3)	56 (96.6)	16 (80.0)	0.017	56 (100)	16 (72.7)	<0.0001
Levofloxacin	72 (92.3)	56 (96.6)	16 (80.0)	0.017	56 (100)	16 (72.7)	<0.0001
Doxycycline	67 (85.8)	50 (86.2)	17 (85.0)	0.894	50 (89.2)	17 (77.2)	0.170
Ertapenem	55 (70.5)	51 (87.9)	4 (20.0)	<0.0001	55 (98.2)	0 (0)	<0.0001
Imipenem	54 (69.2)	51 (87.9)	3 (15.0)	<0.0001	54 (96.4)	0 (0)	<0.0001
Meropenem	55 (70.5)	51 (87.9)	4 (20.0)	<0.0001	55 (98.2)	0 (0)	<0.0001
Minocycline	45 (57.6)	34 (58.6)	11 (55.0)	0.777	34 (60.7)	11 (50.0)	0.389
Piperacillin-tazobactam	70 (89.7)	56 (96.6)	14 (70.0)	0.001	55 (98.2)	15 (68.1)	<0.0001
Ticarcillin-clavulanic acid	73 (93.5)	57 (98.3)	16 (80.0)	0.004	56 (100)	17 (77.2)	<0.0001
Trimethoprim-sulfamethoxazole	70 (89.7)	54 (93.1)	16 (80.0)	0.096	55 (98.2)	15 (68.1)	<0.0001
Tigecycline	18 (23.0)	12 (20.7)	6 (30.0)	0.394	11 (19.6)	7 (31.8)	0.251
Colistin	9 (11.5)	8 (13.8)	1 (5.0)	0.289	9 (16.0)	0 (0)	<0.0001

*ESBL, extended-spectrum β-lactamase*.

### Characterization of Resistance Genes

Among the 58 ESBL producers, the predominant enzyme was CTX-M (57/58, 98.3%), followed by TEM (43/58, 74.1%). *bla*_CTX−M−14_ (44/58, 75.9%) was the most frequently found genotype in ESBL-producers, together with *bla*_CTX−M−15_ (15/58, 25.9%) and *bla*_CTX−M−55_ (8/58, 13.8%), which were relatively less common. All TEM enzymes were encoded by *bla*_TEM−1_. No *bla*_CTX−M_ _(−2, −8, −25group)_, *bla*_GES_, *bla*_VEB_, *bla*_OXA(−2, −10group)_, or *bla*_PER_ genes were found. Although *bla*_SHV_ was detected in 40 strains and *bla*_OXA(−1group)_ in eight strains, the sequencing results demonstrated that these genes were *bla*_SHV−1_, *bla*_SHV−11_, and *bla*_OXA−1_, which belonged to β-lactamase genes rather than ESBL genes. It was also worth noting that 44 of 58 ESBL-producers harbored two or more ESBL genes.

The KPC enzyme is the only carbapenemase produced by isolates resistant to carbapenem, and all KPC enzymes were encoded by *bla*_KPC−2_, with no other carbapenemase genes detected in our study.

### Multilocus Sequence Typing

**Figure 1:**
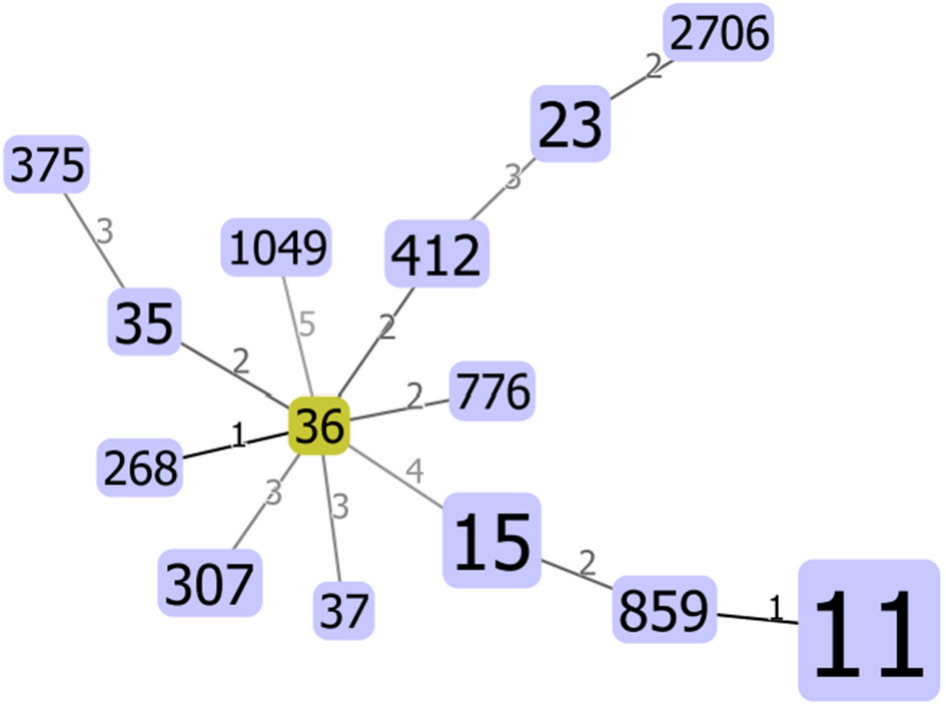
Minimum spanning tree (MST) constructed based on diversity of seven housekeeping genes of *Klebsiella pneumoniae*. The area of each circle corresponded to the prevalence of the sequence type (ST) in the multilocus sequence typing (MLST) data of this study.

Seventeen STs, including three new STs, were identified in 78 *K. pneumoniae* isolates, among which the most principal STs were ST11 (50/78, 64.1%), followed by ST15 (7/78, 9.0%) and ST23 (3/78, 3.8%). All STs were clustered into one non-overlapping group (Figure 1).

## Discussion

As a critical nosocomial pathogen, *K. pneumoniae* is one of the most common causative factors of BSIs, and the worldwide dissemination of antimicrobial-resistant *K. pneumoniae*, especially β-lactam and carbapenem-resistant *K. pneumoniae*, has attracted global attention due to the limited treatment options and high mortality (19–21). Alongside its high antimicrobial-resistant rate, *K. pneumoniae* is also increasingly associated with high virulence, which is called hypervirulent *K. pneumoniae* and can cause severe infections, including liver abscesses and bacteremia (22). Furthermore, *K. pneumoniae* is the most frequent pathogen responsible for ICU-BSIs, representing about 36.8% of all the ICU-BSIs in our hospital. Since ICU patients are predisposed to bacteremia, which can exert a negative impact on the prognosis (23), our study focused on ICU-BSIs caused by *K. pneumoniae* and intended to elucidate antibiotics susceptibility, resistance gene distribution, and STs of these *K. pneumoniae* so that clinicians can administer timely and appropriate antibiotics to improve the prognosis.

Consecutive ICU-BSI *K. pneumoniae* isolates in Shanghai from January 2016 to December 2019 were enrolled in this study. Comparing with the previous study, which investigated the molecular epidemiology of BSI *K. pneumoniae* from comprehensive source collected between 2012 and 2015, this recent research demonstrated that ICU-BSI *K. pneumoniae* isolates harbored extremely higher resistance rate to nearly all commonly used antibiotics, such as ceftazidime, cefepime, cefotaxime, amikacin, gentamicin, tobramycin, aztreonam, ciprofloxacin, levofloxacin, piperacillin-tazobactam, imipenem, meropenem, and trimethoprim-sulfamethoxazole (19). It is also worth noting that the proportion of MDR (23/78, 29.5%) and XDR (48/78, 61.5%) in this study is higher than that in other literature (24). Although ICU-BSI *K. pneumoniae* acquired great resistance to most antibiotics, it was relatively susceptible to tigecycline and colistin, which supported them as potential choices for empirical treatment of ICU-BSI caused by *K. pneumoniae*. However, in critically ill patients, frequent administration of colistin and tigecycline were considered as risk factors for colistin and tigecycline-resistant *K. pneumoniae* BSIs, respectively (25). Therefore, some new antibiotics have been developed, such as ceftazidime-avibactam, which can improve the prognosis of bacteremia associated with carbapenem-resistant *K. pneumoniae* deprived of metallo-β-lactamase (26–28) and can decrease the usage of tigecycline and colistin to slow down the evolution of antibiotic resistance.

In this study, the proportion of ESBL-producing *K. pneumoniae* isolated from ICU patients with BSI was 74.4%, much higher than our previous study (27.5%) (19), and also exceeded the proportion in Hong Kong (12%), Thailand (27.4%), and American (15.5%) (29–31). Despite the distinct ESBL-producing rate, the main type of ESBLs in *K. pneumoniae* was the CTX-M enzyme in our study, consistent with the global trend (32). Although *bla*_CTX−M−15_ was the dominant gene type in most regions worldwide, including India, Iran, and Lebanon (20, 33, 34), *bla*_CTX−M−14_ was the most prevalent ESBL gene found in our study, followed by *bla*_CTX−M−15_ and *bla*_CTX−M−55_, which indicated that the distribution of *bla*_CTX−M_ genes may be geographically different. Similarly, the rate of carbapenemase-producer in ICU-BSI *K. pneumoniae* (71.8%) was higher than that in Taiwan (25%), Athens (60.7%), and Greece (62.3%) (34–36). All carbapenemase-producing ICU-BSI *K. pneumoniae* isolates harbored blaKPC-2, similar to the study conducted in central China (37). *K. pneumoniae* with KPC-2, comparing with NDM-1, was more resistant to amikacin and fosfomycin but more susceptible to trimethoprim/sulfamethoxazole, and there were fewer appropriate treatment choices for KPC-2-producing *K. pneumoniae* (38). According to the consequence of MLST, ST11 was the most predominant ST, and 90% of them possessed the *bla*_KPC−2_ gene, coinciding with other studies in China (6, 37). The similarity of antibiotics resistance pattern and the prevalence of ST11 suggested that the nosocomial clonal dissemination of KPC-2-producing ST11 *K. pneumoniae* happened in ICU patients (39), indicating that current prevention strategies against *K. pneumoniae* in ICU should be adjusted and improved (40). Moreover, ST11 was also dominant among hypervirulent carbapenemase-producing *K. pneumoniae*, which was frequently associated with severe infections (41, 42). Thus, we are likely to pay attention to the virulence factors of ICU-BSI *K. pneumoniae* and find out whether the convergence of carbapenemase production and hypervirulence will exert a negative effect on the outcomes of ICU patients with BSI in our following studies. It is noteworthy that two freshly new STs in our study harbored both ESBL and carbapenemase genes, suggesting the widespread of the antibiotic resistance genes.

## Conclusion

In conclusion, this retrospective study focused on drug susceptibility and molecular epidemiology of *K. pneumoniae* from ICU patients with BSI in Shanghai. Our data demonstrated that ICU-BSI *K. pneumoniae* isolates were highly resistant to clinically common antibiotics, except for tigecycline and colistin. Thus, it would be relatively appropriate to select tigecycline and colistin for empirical treatment. MLST and genetic analysis showed that nosocomial clonal dissemination of KPC-2-producing ST11 had already appeared in ICUs. Meanwhile, *bla*_CTX−M−14_ and *bla*_KPC−2_ were confirmed as the most prevalent ESBL and carbapenemase genes, respectively. These alert us that rational administration of antibiotics and regular surveillance of the molecular epidemiology of pathogens were urgent to impede the dissemination of such highly antimicrobial-resistant and mortal pathogens.

## Data Availability Statement

The raw data supporting the conclusions of this article will be made available by the authors, without undue reservation.

## Ethics Statement

This study was approved by Ethics Committee of Ruijin Hospital affiliated to Shanghai Jiaotong University School of Medicine. The Review Board waived request for informed consent because our study only put emphasis on bacteria and exerted no effect on patients.

## Author Contributions

LH, QZ, and EC contributed to the conceptualization of the study. SX and TC were involved in data curation. TC, HW, and QC performed the formal analysis. EC contributed to funding acquisition. EC, ZY, and LH collected resources. TC involved in writing and original draft preparation. SX helped in writing, review, and editing. All authors contributed to the article and approved the submitted version.

## Funding

The authors acknowledge the support of the Shanghai Jiao Tong University School of Medicine Multicenter Clinical Research Program (DLY201803).

## Conflict of Interest

The authors declare that the research was conducted in the absence of any commercial or financial relationships that could be construed as a potential conflict of interest.

## Publisher's Note

All claims expressed in this article are solely those of the authors and do not necessarily represent those of their affiliated organizations, or those of the publisher, the editors and the reviewers. Any product that may be evaluated in this article, or claim that may be made by its manufacturer, is not guaranteed or endorsed by the publisher.
